# Exercise, Cellular Senescence, and Cancer: Novel Perspectives on Functional Aging Through Block Strength Training in Older Adults—A Narrative Review

**DOI:** 10.3390/biomedicines14040875

**Published:** 2026-04-11

**Authors:** Rodrigo L. Castillo, Emilio Jofré-Saldía, Daniela Cáceres-Vergara, Georgina M. Renard, Esteban G. Figueroa

**Affiliations:** 1 Departamento de Medicina Interna Oriente, Facultad de Medicina, Universidad de Chile, Santiago 7500922, Chile; rodrigouch@uchile.cl; 2 Unidad de Paciente Crítico, Hospital del Salvador, Santiago 7500922, Chile; 3 Escuela de Ciencias de la Actividad Física, Facultad de Ciencias de la Rehabilitación y Calidad de Vida, Universidad San Sebastián, Santiago 7510157, Chile; 4 Centro de Investigación Biomédica y Aplicada (CIBAP), Escuela de Medicina, Facultad de Ciencias Médicas, Universidad de Santiago de Chile, Santiago 9170022, Chile; daniela.caceres.ve@usach.cl (D.C.-V.); georgina.renard@usach.cl (G.M.R.); 5 Escuela de Obstetricia, Facultad de Ciencias Para el Cuidado de la Salud, Universidad San Sebastián, Santiago 7510157, Chile

**Keywords:** cellular senescence, aging, block strength training, older adults, cancer prevention

## Abstract

Population aging has markedly increased the burden of cancer in older adults, in whom frailty, sarcopenia, and reduced physiological reserve limit tolerance to treatment and worsen clinical outcomes. Aging is accompanied by progressive functional decline and by biological processes such as cellular senescence, characterized by irreversible cell cycle arrest, chronic low-grade inflammation, and impaired immune surveillance. The accumulation of senescent cells and the persistence of a senescence-associated secretory phenotype contribute to tissue dysfunction and generate a microenvironment that favors tumor initiation and progression. Physical exercise has been associated with attenuation of inflammation, improvements in metabolic and immune function, and with lower levels of senescence-related biomarkers. Although aerobic exercise has been extensively studied in this setting, resistance training holds relevance for older adults due to its capacity to counteract sarcopenia, preserve muscle strength and power, and sustain functional independence. Structured and periodized approaches to resistance exercise may further enhance these benefits by delivering targeted stimuli aligned with age-related physiological deficits. Block strength training (BST), a periodized model that concentrates training adaptations into sequential phases of maximal strength, power, and muscular endurance, has demonstrated consistent improvements in functional performance and reductions in frailty risk in community-dwelling older adults. BST improves physical function. It may also influence biological processes related to aging and cancer; however, mechanistic evidence specific to BST remains to be established. We hypothesized that the exercise in block as a targeted, a structured and physiologically grounded resistance training intervention highlights the potential of BST to promote functional aging and healthy. In the case of cancer biology, and the environment near to tumour, the relationship between aging mechanisms in older adults and controlled exercise effects are currently in advance, but mechanistic trials are still lacking. Finally, we propose a novel training method, structured and personalized, that could impact different clinical outcomes in older patients with cancer.

## 1. Introduction

Cancer is one of the leading causes of morbidity and mortality worldwide, and its incidence increases exponentially with age. In 2020, it was estimated that there were 19.3 million new cancer cases globally, with nearly 64% of these diagnoses occurring in individuals aged 60 or older [1]. Projections of cancer incidence indicate that by 2040, the number of new cases among older adults will reach 20.7 million, with 12.7 million deaths [1]. The progressive aging of the population has led to an increasing proportion of cancer patients being older adults. Cancer in older adults represents a growing challenge. In women, menopause-related changes and higher frailty prevalence may further influence vulnerability, but most mechanistic evidence remains mixed-sex. A key factor in this context is frailty, a geriatric syndrome of systemic vulnerability. Frailty is a state of susceptibility to poor homeostatic resolution following a stressful event and is the result of cumulative deterioration in multiple physiological systems [2]. Approximately 10% of individuals over the age of 65 exhibit frailty, with this percentage increasing between 25% and 50% in individuals over 85 years old [3]. Frailty is highly common among elderly cancer patients; in fact, more than half of older adults with cancer exhibit pre-frailty or frailty, which is associated with a higher risk of treatment intolerance, postoperative complications, and mortality [3,4]. These data underscore the importance of addressing cancer in the context of aging from an integrated geriatric perspective.

Furthermore, the evidence supports the crucial role of physical exercise in promoting healthy aging. Regular physical activity (PA) prevents much of the functional decline typical of older age and the burden of chronic diseases, acting as a true “anti-aging medicine” [5]. Exercise has been shown to improve cardiovascular health, maintain muscle mass and balance, and reduce the risk of prevalent conditions in older adults such as hypertension [6], obesity, diabetes [7], and even certain cancers [8]. In addition, habitual PA provides significant psychological and cognitive benefits; for example, moderate PA sessions can alleviate anxiety and improve mood acutely, and in the long term, they are associated with less cognitive decline and a lower risk of depression in older adults [9,10]. At the cellular and molecular level, an active lifestyle counteracts some of the biological mechanisms of aging: multiple studies suggest that regular exercise reduces senescent cell burden across tissues [11,12] and attenuates the chronic low-grade inflammation associated with aging [13]. This inflammatory process is characterized by the continuous activation of the immune system without the presence of a clear acute infection. It is often subtle and can be caused by various factors, including obesity, persistent infections, autoimmune diseases, and environmental toxins. Moreover, this is a significant risk factor for cancer development, as it influences multiple pathways involved in tumor initiation, progression, and metastasis [14].

The concept of functional aging emphasizes the ability of older adults to maintain independence and physical, mental, and social functioning, irrespective of chronological age (WHO, 2020 [15]). This entails prioritizing the extension of healthy life (increasing healthy life expectancy) over mere longevity. In fact, there is a significant gap at the population level—estimated at about 9 years on average—between life expectancy and years of healthy life free of disability, meaning that many older adults spend their final years with chronic diseases or functional limitations [16]. Therefore, current strategies aim to “compress” this age-associated morbidity by preventing frailty and maintaining functional capacity for as many years as possible. Ultimately, the biological changes underlying aging, such as cellular senescence, play a decisive role in age-related functional decline and in the pathogenesis of cancer [17].

Regarding the type of physical activity, strength training has been described as having a significant benefit in reducing frailty and sarcopenia events in cancer patients [18]. In terms of outcomes, the perception of quality of life has an impact on symptom improvement [19]. Furthermore, structured physical activity that combines strength and aerobic exercise has beneficial effects on reducing frailty [20].

In this review, we integrate current knowledge on cellular senescence, exercise biology, and sarcopenia with a specific focus on older adults, and propose block strength training (BST) as a biologically informed resistance training model. In this view, the burden of cancer disproportionately affects older adults, underscoring the need for interventions to address functional limitations and prevent further health consequences of cancer and its treatment [21]. Although direct mechanistic and clinical evidence specifically addressing BST in the context of cellular senescence and cancer remains limited, this model is presented here as a biologically plausible and hypothesis-generating framework grounded in established resistance training literature.

Unlike previous reviews that address exercise or sarcopenia in isolation, we explicitly link BST with senescence-related pathways and cancer-relevant outcomes, outlining a translational framework that connects molecular hallmarks of aging with functional and clinical endpoints in this population [22,23]. This work attempts to substantiate the hypothesis of the likely beneficial effect of “BTS” in cancer patients.

These novel associations between BTS and clinical effectiveness in training may provide the basis for future intervention protocols in older patients.

## 2. Methodological Considerations

The purpose of this review is to integrate cellular senescence, exercise biology, sarcopenia, and cancer risk, proposing block strength training (BST) as a biologically informed resistance training model.

A focused, non-systematic literature search was conducted in PubMed, Scopus, and Web of Science to identify relevant original research articles and reviews published between 2000 and 2025. The search was updated prior to manuscript submission to ensure inclusion of the most recent available evidence. Combinations of the following keywords were used: “cellular senescence”, “senescence-associated secretory phenotype”, “exercise”, “physical activity”, “resistance training”, “strength training”, “block strength training”, “periodized training”, “sarcopenia”, “older adults”, “postmenopausal women”, “cancer prevention”, “aging”, and “inflammaging”. Only articles published in English were considered

Articles were selected based on their conceptual relevance to the integration of senescence biology, resistance training adaptations, and cancer-related mechanisms in older adults. Priority was given to mechanistic studies, clinical trials, meta-analyses, and high-quality narrative or systematic reviews. When foundational mechanistic evidence was required, seminal studies published prior to 2000 were exceptionally included.

This narrative review was structured in accordance with the SANRA (Scale for the Assessment of Narrative Review Articles) framework to ensure methodological transparency and scientific rigor in the selection and synthesis of the evidence [24].

## 3. Cellular Senescence in Aging and Cancer

Cellular senescence is a state of permanent cell cycle arrest in response to cellular damage or stressors such as DNA damage, telomere shortening, or oncogenic activation. This biological process strongly contributes to the development of chronic diseases, including cancer [25]. Mechanistically, this irreversible arrest is mediated by p53 protein and cyclin-dependent kinase inhibitor 1A (p53/p21^CIP1^) and/or tumor suppressor pathway involving cyclin-dependent kinase inhibitor 2a and retinoblastoma protein (p16^INK4a/Rb^) [26]. Despite ceasing to divide, senescent cells remain metabolically active and develop a senescence-associated secretory phenotype (SASP), characterized by increased production of pro-inflammatory bioactive factors, including cytokines, chemokines, growth factors, proteases, and other extracellular matrix components [27,28]. These changes are accompanied by increased lysosomal content and activity of the acidic β-galactosidase enzyme (SA-β-gal), whose histochemical detection is a classic marker of senescent cells [26]. Likewise, senescent cells commonly exhibit overexpression of cell cycle inhibitors such as p16^INK4a^ and p21^CIP1^, reflecting their state of permanent proliferative arrest [29].

Senescent cells tend to accumulate in tissues as aging progresses, due to repeated exposure to damaging factors throughout life [29]. Indeed, studies in model organisms have shown that senescent cells progressively increase with age and functionally contribute to age-related decline [30]. The growing presence of these dysfunctional cells is considered a fundamental driver of aging and age-associated tissue dysfunction, to the extent that cellular senescence is recognized as one of the key hallmarks of aging [31]. A central mechanism by which senescent cells promote aging is through the SASP: factors secreted by senescent cells sustain a state of chronic low-grade inflammation, known as “inflammaging” [32], observed in aged tissues. This persistent pro-inflammatory environment, driven by cytokines such as interleukin 6 (IL-6), interleukin 1 beta (IL-1β), and tumor necrosis factor alpha (TNF-α) secreted by senescent cells, progressively disrupts tissue homeostasis and favors functional decline of organs and systems [33,34]. In addition, immune surveillance of senescent cells declines with age: chronic inflammation and other mechanisms allow senescent cells to evade elimination by natural killer (NK) cells and cytotoxic T lymphocytes, leading to their preferential accumulation in aged tissues [35]. This accumulation, together with the resulting SASP, generates a vicious cycle of inflammation and tissue damage that contributes to aging and increases vulnerability to chronic diseases [35].

**Figure 1:**
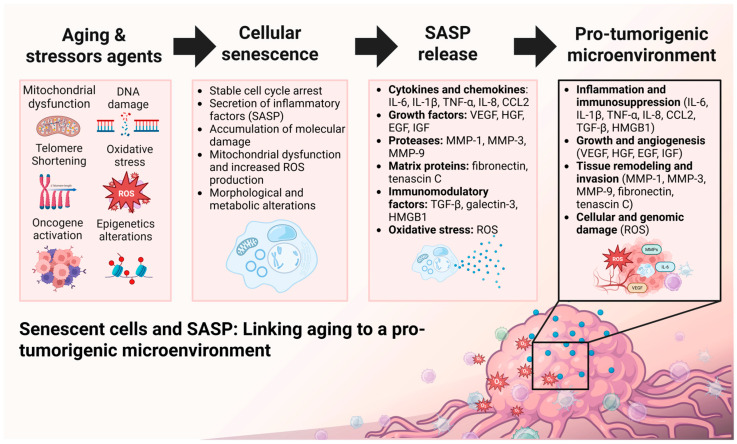
Cellular senescence and age-associated tumorigenic microenvironment. Schematic representation of stressors that induce cellular senescence (e.g., DNA damage, telomere shortening, oxidative stress, oncogenic activation) and the development of a senescence-associated secretory phenotype (SASP). Accumulation of senescent cells and SASP-related signaling contribute to chronic inflammation, tissue remodeling, and a microenvironment permissive to tumor development in aging tissues. Abbreviations: Interleukin 6 (IL-6), Interleukin 1 beta (IL-1β), Tumor necrosis factor alpha (TNF-α), Interleukin 8 (IL-8), Chemokine (C-C motif) ligand 2 (CCL2), Reactive oxygen species (ROS), Senescence-associated secretory phenotype (SASP), Matrix metalloproteinases (MMP), Vascular endothelial growth factor (VEGF), Hepatocyte growth factor (HGF), Epidermal growth factor (EGF), Insulin-like growth factor (IGF), Transforming growth factor beta (TGF-β), High-mobility group box 1 (HMGB1).

Cellular senescence plays a complex and dual role in cancer biology. On the one hand, during early life it acts as a tumor-suppressive mechanism: when a cell experiences oncogenic damage or severe stress, entry into senescence prevents uncontrolled proliferation and thereby inhibits tumor formation [36]. Certain SASP factors may initially contribute to immune-mediated clearance of damaged cells; for instance, cytokines produced by senescent cells can recruit immune cells that recognize and eliminate premalignant cells before progression [37,38]. On the other hand, in the context of advanced age, senescence becomes detrimental. The persistent presence of senescent cells in the tissue microenvironment over prolonged periods promotes tumorigenesis: chronically secreted SASP factors induce inflammation and tissue remodeling that favor tumor development and progression [36]. Studies have shown that when senescent cells accumulate unchecked, their SASP can directly stimulate the growth of emerging malignant cells or create an immunosuppressive environment that allows cancer cells to evade immune surveillance [39,40,41]. Thus, although the induction of senescence initially serves as a protective mechanism against cancer, the long-term accumulation of senescent cells in aged tissues ultimately contributes to age-associated pathologies—including fibrosis, degenerative diseases, and cancer—through chronic inflammation and pro-tumorigenic alterations of the tissue microenvironment. This dual behavior illustrates the “double-edged sword” nature of cellular senescence in carcinogenesis (Figure 1).

At the molecular level, the SASP phenotype of senescent cells promotes multiple processes associated with cancer initiation and progression. Pro-inflammatory cytokines released (such as IL-6, IL-1β, and TNF-α, among others) generate an inflammatory microenvironment that not only damages surrounding healthy tissue but can also induce genotoxic stress in neighboring cells, facilitating malignant transformation over time [35]. SASP factors also help “prepare the soil” for cancer by supporting key processes of tumor development: for example, they promote angiogenesis and drive stromal changes that facilitate epithelial–mesenchymal transition (EMT) and cancer cell invasion. In particular, enzymes such as matrix metalloproteinases (MMPs) secreted by senescent cells degrade and remodel the extracellular matrix [28,42], weakening normal tissue architecture and creating space for tumor expansion and invasion within the tumor microenvironment [43]. Certain SASP factors can reprogram nearby cells toward tumor-promoting states or induce senescence in initially healthy cells, thereby generating additional senescent cells within the tumor microenvironment [39,44]. Moreover, the SASP interferes with antitumor immune responses through the chronic release of inflammatory mediators that exhaust cytotoxic lymphocytes and NK cells [44,45]. In parallel, SASP components such as GDF15 and pro-inflammatory cytokines can skew or divert macrophages and other innate immune cells toward immunosuppressive phenotypes, thereby facilitating tumor immune escape [46,47]. Another important link is telomere shortening, which occurs with each cell division and triggers replicative senescence as a protective barrier against uncontrolled proliferation. However, when a precancerous cell manages to bypass this brake—by activating telomerase to maintain telomere length—it acquires unlimited replicative capacity, while critically short or dysfunctional telomeres can drive genomic instability (chromosomal fusions and aberrations) that further increase the risk of malignant transformation [29,45].

Multiple observations support the relevance of cellular senescence in humans. For example, expression of the marker p16^INK4a^ increases with age in numerous tissues [48]; in human T lymphocytes, p16^INK4a^ levels correlate strongly with biological age [49]. This finding suggests that the burden of senescent cells reflects human aging. Given the deleterious impact of accumulated senescent cells, substantial interest has emerged in strategies to manage this cellular burden. Preclinical studies in animal models have causally demonstrated the role of senescent cells in aging and cancer: for instance, in transgenic mice engineered to selectively eliminate senescent cells, periodic clearance of senescent cells led to marked improvements in health, extended median lifespan, and delayed the onset of age-associated spontaneous tumors [50]. Conversely, experimental transfer of a small number of senescent cells into young mice is sufficient to induce aging-like features, reinforcing the concept that surpassing a threshold of senescent cell burden triggers age-related pathology [51,52]. In recent years, senolytic drugs (which selectively eliminate senescent cells) and senomorphic agents (which inhibit or modulate the SASP without killing the cells) have been developed as potential interventions against age-related diseases [52,53]. Importantly, early human trials are already underway: small initial clinical studies have shown encouraging results, demonstrating functional improvements in patients following reductions in senescent cell burden, and multiple larger clinical trials are currently in progress [54,55]. Overall, therapeutic targeting of senescent cells (either through selective elimination or attenuation of their inflammatory SASP profile) emerges as a promising strategy to delay aging and prevent age-related pathologies, including cancer. Nevertheless, physical exercise also plays an important role as a non-pharmacological approach or as an adjuvant strategy in the modulation of cellular senescence.

## 4. Exercise as a Modulator of Cellular Senescence

Regular exercise is an effective lifestyle intervention for slowing multiple aspects of biological aging. It may attenuate several hallmarks of aging, including chronic inflammation and the accumulation of senescent cells, through widespread systemic and cellular adaptations [12,56,57]. For example, long-term physical training has been shown to reduce oxidative stress and inflammation while enhancing DNA repair mechanisms and proteostasis in mixed-sex cohorts [58,59]. However, these studies did not provide sex-stratified analyses, and therefore sex-specific effects in older women require further investigation.

Exercise is known to engage pathways such as AMPK signaling, which promotes cellular metabolic health and can inhibit senescence-related processes [60]. Together, these combined effects help counteract age-related damage and preserve tissue functionality.

There is growing evidence indicating that exercise reduces the burden of senescent cells in both animal models and humans. In mice, aerobic endurance training has been associated with a decrease in cellular senescence markers; for instance, four weeks of treadmill running significantly reduced the expression of p16^INK4a^ and p21^CIP1^ in skeletal muscle [60]. In aged rats, a two-month weighted ladder-climbing training protocol suppressed the accumulation of senescent fibro-adipogenic progenitors (FAPs), reduced SASP factor secretion, and increased cluster of differentiation 8 positive T lymphocyte (CD8^+^ T) cell infiltration in muscle [12,61]. Human studies support these findings. A clinical trial in older adults showed that a structured 12-week exercise program significantly reduced circulating senescence-associated biomarkers, including p16^INK4a^, p21^CIP1^, and TNF-α expression in T lymphocytes, as well as multiple SASP proteins in plasma [12]. This provides in vivo evidence that exercise can reduce circulating senescence-associated biomarkers; whether this reflects reduced senescent cell burden versus changes in immune cell composition or SASP signaling remains to be clarified. However, whether these reductions reflect a true decrease in senescent cell burden or are driven by changes in immune cell composition, altered secretory activity, or systemic inflammatory modulation remains to be elucidated. Therefore, circulating biomarkers should be interpreted as indicators of senescence-associated systemic signaling rather than direct measures of senescent cell accumulation.

Exercise may contribute to the preservation of telomere length and telomerase activity, which has been associated with protection against replicative senescence. Observational studies indicate that physically active adults have longer leukocyte telomeres than their sedentary counterparts [62,63]. For example, older women with higher levels of moderate-to-vigorous PA exhibited significantly longer telomeres [62]. These effects are partly explained by exercise-induced reductions in oxidative stress and inflammation, which slows telomere erosion [64]. By helping to maintain telomere integrity, regular exercise could delay replicative senescence in dividing cell populations.

Exercise further reduces the chronic low-grade inflammation associated with aging, in part by decreasing pro-inflammatory SASP factors secreted by senescent cells. Physically active older adults show significantly lower levels of cytokines such as IL-6, TNF-α, and C-reactive protein compared with sedentary individuals [65,66,67]. One study demonstrated that consistently active older adults had lower tissue expression of IL-6 in the colonic mucosa than sedentary controls [68]. This suppression of the SASP promotes a less inflammatory tissue microenvironment and one that is less permissive to tumor growth [11,69,70].

**Figure 2:**
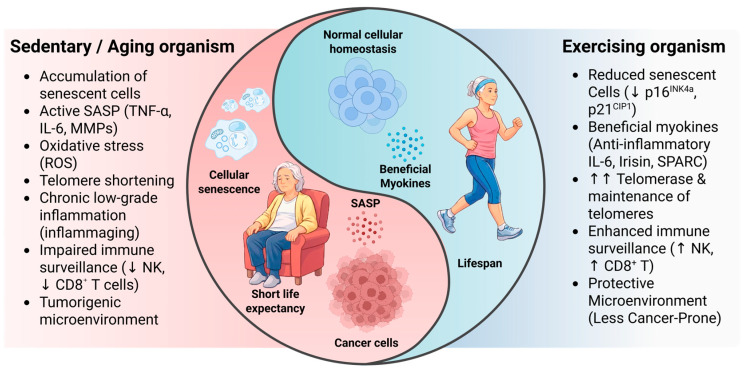
Sedentary versus physically active aging: impact on senescence-related pathways. Conceptual comparison between sedentary aging and regular physical activity in older adults. The sedentary state is associated with increased senescence-related markers, chronic inflammation, and impaired immune function. In contrast, regular exercise is associated with lower inflammatory signaling, preserved immune surveillance, and a less tumor-promoting microenvironment. Arrows indicate direction of change: ↑ increase; ↓ decrease. Abbreviations: senescence-associated secretory phenotype (SASP), tumor necrosis factor alpha (TNF-α), interleukin-6 (IL-6), matrix metalloproteinases (MMPs), reactive oxygen species (ROS), natural killer cells (NK), Cluster of differentiation 8 positive T lymphocyte (CD8^+^ T), cyclin-dependent kinase inhibitor 1A (p21^CIP1^), cyclin-dependent kinase inhibitor 4A (p16^INK4a^).

Contracting skeletal muscle releases myokines such as IL-6 (in its anti-inflammatory role), irisin, and secreted protein acidic and rich in cysteine (SPARC), which exert systemic effects on inflammation, tissue regeneration, and immune modulation [71]. Exercise-induced IL-6 stimulates the production of IL-1ra and IL-10 and inhibits TNF-α, contributing to the resolution of inflammation [72,73]. Irisin has also been associated with activation of AMP-activated protein kinase (AMPK) and sirtuin 1 (SIRT1), pathways involved in cellular longevity [74]. Moreover, exercise enhances immune surveillance by increasing the activity of NK cells and cytotoxic T lymphocytes, thereby facilitating the clearance of senescent or emerging tumor cells [75,76,77]. This interaction between skeletal muscle and the immune system, the so-called “myokine network”, constitutes a key mechanism through which exercise may promote healthy longevity and cancer prevention. Myokines are signaling molecules released specifically by skeletal muscle during contraction, whereas exerkines refer to a broader group of exercise-induced factors derived from multiple tissues, including muscle, adipose tissue, and other organs [78]. This complex network constitutes a key molecular mechanism for promoting healthy longevity and preventing cancer, acting by mitigating chronic inflammation (inflammaging), optimizing immune surveillance, and regulating cellular senescence profiles. These mechanisms are visually contrasted in Figure 2, which highlights the biological and functional divergence between sedentary and physically active aging in older adults.

## 5. Sarcopenia, Strength Training, and Functional Aging in Older Adults

Sarcopenia is defined as a progressive and generalized skeletal muscle disorder characterized by the loss of muscle mass and, more critically, muscle strength, leading to an increased risk of falls, physical disability, and premature mortality [79]. This condition represents a major determinant of functional decline and reduced quality of life in later life [80]. In older women, sarcopenia is further aggravated by menopause-related hormonal changes, particularly the drop in estrogen levels, which accelerates musculoskeletal deterioration and predisposes women to greater losses in physical performance and autonomy compared with age-matched men [81].

Beyond its functional consequences, sarcopenia exerts a substantial metabolic impact. Reductions in skeletal muscle mass impair insulin sensitivity and glucose homeostasis, increasing the risk of metabolic syndrome and type 2 diabetes in aging populations [82]. In parallel, age-related declines in muscle strength and, especially, muscle power—defined as the capacity to generate force rapidly—reduce physiological reserve and resilience, increasing vulnerability to stressors and the likelihood of adverse events such as falls or acute injuries [22]. From a functional perspective, muscle power is particularly relevant. Its impairment is strongly associated with difficulties in daily activities that require rapid force production, such as rising from a chair, stair negotiation, or balance recovery following perturbations [83].

Importantly, age-related neuromuscular decline does not occur uniformly across physical capacities. As emphasized by Hunter et al. (2019) [82], declines in muscular power typically exceed declines in maximal muscle strength with aging, reflecting combined alterations in muscle morphology, neural drive, and contractile velocity. This disproportionate decline in power has critical functional consequences, as many activities of daily living depend more on rapid force production than on maximal strength alone [82]. This framework reinforces the concept that exercise interventions should prioritize specific neuromuscular capacities rather than treating muscle function as a single, homogeneous outcome. Intervention studies have demonstrated that muscle power training is not only feasible but particularly effective for enhancing functional performance in older adults. For instance, explosive or high-velocity resistance training protocols have been shown to significantly improve rapid force production, muscle power, and functional outcomes such as chair-rise performance and gait speed in older individuals [84,85]. More recent research has further explored structured training strategies aimed at optimizing skeletal muscle performance in aging populations, such as the DART (Developmental Adaptations in Response to Training) randomized trial framework [86]. In addition, large-scale meta-analytic evidence indicates that resistance training interventions consistently improve physical performance, muscle strength, and functional capacity in older adults [87]. Together, these findings support the prioritization of muscle power development in aging populations to preserve autonomy and mitigate frailty risk.

From a pathophysiological standpoint, sarcopenia is closely intertwined with cardiometabolic deterioration and systemic biological dysregulation [88,89]. Sarcopenia-associated adipokine overexpression can promote oxidative stress, facilitating low-density lipoprotein oxidation, impaired cholesterol efflux, and collagen fiber aggregation within fibroatheroma plaques [90,91]. Collectively, these processes exacerbate endothelial dysfunction and accelerate atherogenesis, increasing the risk of multifactorial cardiovascular disease, including ischemic heart disease, arrhythmias, and heart failure [88,92]. Recent evidence suggests an independent association between sarcopenia and coronary artery calcification, a marker of advanced atherosclerosis and a predictor of future cardiovascular events [93].

From a biological aging perspective, sarcopenia should be understood not only as a functional phenotype but also as a systemic state characterized by chronic inflammation, oxidative stress, and impaired immune competence [32,94]. These features overlap with senescence-associated signaling pathways and may contribute to a permissive microenvironment for age-related disease progression, including cancer [95,96]. Consequently, interventions aimed at preserving skeletal muscle function and improving redox–inflammatory balance may have relevance beyond mobility outcomes, potentially influencing senescence burden and immune surveillance in older women [94]. Also, clinical evidence in some meta-analysis showed that in older adults with sarcopenia, high or moderate certainty evidence showed that resistance exercise with or without nutrition and the combination of resistance exercise with aerobic and balance training were the most effective interventions for improving quality of life in older adults of both sexes [97].

In this context, resistance training emerges as the most effective preventive and therapeutic strategy to counteract sarcopenia and its functional consequences. Skeletal muscle retains considerable plasticity even at advanced ages, and progressive resistance training consistently improves muscle strength, muscle power, and, to a lesser extent, muscle hypertrophy in older adults [22]. In older women, regular engagement in resistance training is associated with reduced frailty risk, improvements in balance and mobility, preservation of bone mineral density, and enhanced health-related quality of life [22]. These adaptations translate into clinically meaningful functional benefits, including improved chair-rise performance, faster gait speed, and better dynamic balance, reflecting functional rather than chronological age.

Regarding the type of training and effects in older patients, resistance protocols not only improve physical parameters but also directly impact on quality of life: (i) preservation of independence: it allows adults to maintain physical, mental, and social functioning regardless of their chronological age [98]; (ii) compression of morbidity: It helps reduce the approximately 9-year gap between life expectancy and healthy life expectancy, preventing later years from being spent with functional limitations [99]; (iii) reduced disease burden: Consistently active individuals have significantly lower risks of cardiovascular disease (27–33% lower), cancer, and other fatal, debilitating illnesses, which are the primary causes of the gap [100].

## 6. Block Strength Training—Concept and Implementation

From the standpoint of age-related functional alterations and underlying biological mechanisms, the principal challenge in promoting functional aging in older adults is not merely encouraging participation in strength programs but organizing training stimuli capable of counteracting declines in strength, power, and effort tolerance while respecting the biological time course of adaptation [22,82]. Aging skeletal muscle is characterized by reduced anabolic sensitivity to mechanical and nutritional stimuli, attenuated activation of mechanistic target of rapamycin complex 1 (mTORC1), progressive mitochondrial dysfunction, increased oxidative stress, and chronic low-grade [101,102]. These alterations are accompanied by impaired mitochondrial dynamics, reduced Peroxisome Proliferator-Activated Receptor Gamma Coactivator 1-alpha (PGC-1α) signaling, and dysregulated autophagic processes, all of which compromise the adaptive capacity of aged muscle [103,104]. Consequently, resistance training in older adults should be conceptualized not only as a neuromuscular intervention but also as a potential modulator of the molecular milieu associated with sarcopenic aging. Importantly, the proposed biological effects of BST discussed in this section are extrapolated from broader resistance training literature and should not be interpreted as BST-specific mechanistic evidence.

Regarding the physiological and molecular basis of BST in older adults, few clinical studies have determined mechanistic effects on inflammation and the modification of cellular components in skeletal muscle [105]. However, emerging evidence indicates that appropriately dosed resistance exercise can reduce circulating biomarkers associated with cellular senescence and modulate systemic inflammatory profiles in humans [12]. Importantly, these findings derive from the broader resistance training literature and not from BST-specific interventions and therefore should be interpreted as biologically plausible rather than directly demonstrated mechanisms. These findings provide a biologically plausible framework to explore whether a structured and sequenced model such as BST may exert effects extending beyond functional performance.

As a controlled-intensity, full-body training model with sustained exposure across blocks, BST may elicit antioxidant adaptations that help manage exercise-induced oxidative stress. Regular training can increase antioxidant enzyme activity—particularly superoxide dismutase (SOD), catalase (CAT), and glutathione peroxidase (GPx)—in skeletal muscle, thereby improving the capacity to neutralize reactive oxygen species (ROS) generated during repeated high-demand contractions and endurance-like workloads [106]. These adaptations have been described in resistance training contexts and should be interpreted as plausible pathways rather than mechanisms directly demonstrated in BST interventions. Such adaptations provide a biologically plausible link between resistance training programming and redox-sensitive pathways relevant to senescence signaling [106].

Accumulation blocks stimulate mitochondrial biogenesis and protein synthesis in slow-twitch muscle fibres, whereas lower-volume intense workloads of the transmutation blocks evoke adaptive modifications in fast-twitch glycolytic and oxidative-glycolytic muscle fibers [105]. From a mechanistic standpoint, such sequencing may allow differential and temporally organized activation of AMPK, mTOR, and PGC-1α pathways, facilitating both metabolic and structural adaptations while limiting excessive systemic stress [103,105]. These mechanistic interpretations are extrapolated from established exercise physiology principles and have not been directly tested within BST-specific protocols.

In this context, BST emerges as a structured approach that translates established physiological principles into a specific methodology aligned with the consequences of sarcopenic aging [22,107]. Compared with traditional non-periodized or generic multi-component programs, BST concentrates adaptations into clearly defined blocks, prioritizing strength and power development that are often undertrained in standard protocols for older adults [22]. Unlike traditional non-periodized or mixed-method resistance training approaches, BST emphasizes a structured sequencing of dominant training stimuli, prioritizing specific neuromuscular capacities rather than their concurrent development, which may be particularly relevant given the disproportionate decline in muscle power relative to strength observed with aging [22,108]. This targeted sequencing may be particularly advantageous in older adults with sarcopenia; in older women, disproportionate power loss and recovery constraints may make sequencing particularly relevant [84,109]. Importantly, BST does not rely on unique molecular mechanisms but on the structured sequencing of training stimuli, which may allow more precise hormetic dosing compared to non-periodized or concurrent models.

**Figure 3:**
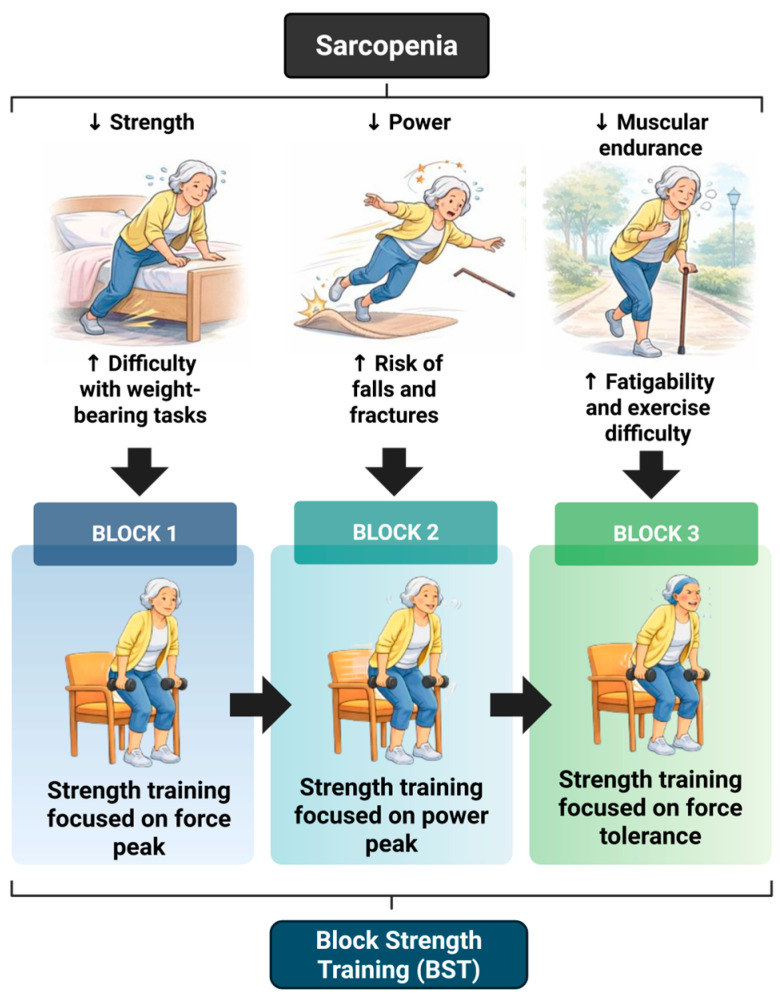
Block Strength Training (BST) model adapted for sarcopenic aging. Schematic overview of a block-periodized resistance training framework composed of sequential phases targeting maximal strength, muscle power, and muscular endurance. The model is designed to address age-related declines in neuromuscular performance and functional capacity in older adults. Arrows indicate direction of change: ↑ increase; ↓ decrease. Adapted from Jofré-Saldía et al., 2025 [108].

By structuring loading, velocity, and volume across blocks, BST operationalizes resistance training principles in a way that is both physiologically specific and clinically feasible in community-dwelling older adults; evidence from trials in older women supports feasibility [108]. BST structures training sequential blocks with dominant adaptive objectives: maximal strength, muscle power, and endurance (Figure 3). This sequence reflects the functional demands of daily life and corresponds to the progressive deficits observed with aging, particularly the accelerated loss of power relative to strength [109]. In addition, it respects the time course of neuromuscular adaptation, which is compromised by slower muscle protein synthesis, reduced anabolic sensitivity, and prolonged recovery in older adults [22].

Unlike models that demand maximal or exhaustive efforts, BST emphasizes sub-maximal efforts (1–3 repetitions in reserve) with a high perceived level of safety. This strategy is effective and better tolerated in older women, who typically exhibit lower resilience to fatigue and a higher risk of dropout when training to failure is prescribed [110,111]. Thus, BST allows for effective neuromuscular stimulation without inducing unnecessary physiological stress [108]. In this sense, precision is not defined solely by load prescription, but by the alignment between external stimulus and individual tolerance to effort. This precision is operationalized through the integration of load, volume, movement velocity, and repetitions in reserve (RIR), enabling dynamic adjustment of the training stimulus according to individual perceptual and physiological responses across training blocks [110,111].

Within each block, variables such as load, volume, velocity, and rest intervals are adjusted to favor the targeted capacity. Strength blocks use moderate-to-high loads with longer rest periods to maximize neural drive; power blocks prioritize movement velocity with submaximal loads; and endurance blocks employ higher repetition schemes to improve effort tolerance [22,108,112] (Table 1). This organization enables systematic modulation of mechanical load, contraction speed, and effort distribution, while respecting the constraints imposed by sarcopenic aging.

The effectiveness of BST is supported by empirical evidence. de Vos et al. [84] and Liu and Latham [21] reported improvements in handgrip strength, gait speed, Timed Up and Go, and other functional tests after nine weeks of block-structured strength training. More recent studies in older women have confirmed these benefits. Importantly, previous findings demonstrate that BST significantly improves handgrip strength, Timed Up and Go, Two-Minute Step Test, 5-Sit-to-Stand, and walking speed in older women [108], with functionally meaningful shifts away from clinical cut-off points associated with frailty and physical vulnerability. Given that BST is structured into three sequential blocks—maximal strength, muscle power, and muscular endurance, it is plausible to hypothesize that each phase may preferentially stimulate distinct yet complementary biological adaptations. The initial strength-oriented block, characterized by moderate-to-high mechanical tension under submaximal conditions, could primarily enhance neuromuscular drive and activate anabolic signaling pathways such as mTORC1, supporting protein synthesis and structural remodeling [113,114]. The subsequent power-focused block, emphasizing high contraction velocity with controlled effort, may promote improvements in motor unit recruitment efficiency, excitation–contraction coupling, and neuromuscular coordination, mechanisms closely associated with the preservation of rapid force production in aging muscle [85,115]. Finally, the muscular endurance block, involving greater metabolic demand with controlled fatigue, could stimulate AMPK-mediated pathways and mitochondrial biogenesis, potentially improving oxidative capacity and metabolic resilience [103].

From a biological perspective, BST could plausibly affect aging-related processes such as oxidative stress, chronic inflammation, and mitochondrial dysfunction. Although these mechanisms have not yet been measured directly in BST interventions, there is robust evidence linking appropriately dosed resistance training to improvements in redox balance, immune function, and cellular surveillance in older adults [94,116]. From an integrative perspective, these functional improvements may also be interpreted through the principle of hormesis [117,118]. Hormesis describes how repeated exposure to low-to-moderate stressors induces adaptive cellular responses that enhance systemic resilience [119]. In aged skeletal muscle, submaximal yet cumulative mechanical stimuli may activate anabolic and metabolic signaling sufficiently to promote adaptation, while avoiding sustained activation of pro-inflammatory and oxidative stress pathways that could impair recovery [119]. Concurrently, BST can be conceptually framed within the General Adaptation Syndrome model, in which each block represents a structured phase of specific stress followed by adequate recovery, enabling progressive superposition of residual training effects [120]. The systematic accumulation of submaximal stimuli—rather than repeated exposure to failure—may optimize the stimulus–adaptation relationship in a tissue characterized by anabolic resistance and metabolic vulnerability. While block-periodized approaches do not invariably outperform multicomponent programs on every outcome, their main strength lies in the specific development of strength and power, which are often undertrained in generic protocols [112]. In this sense, BST does not replace other modalities; rather, it represents a precision strategy that aligns training stimuli with the biological and functional deficits characteristic of sarcopenia. In this sense, BST does not represent a superior model per se, but rather a structured and potentially more tolerable approach through which established resistance training adaptations can be delivered in older adults [22,108].

From a translational perspective, BST provides a structured framework to operationalize knowledge of aging biology into meaningful functional adaptations. Although these molecular mechanisms have not yet been directly tested under a BST framework, the coherence between the magnitude of functional improvements observed [108] and established principles of adaptive stress biology suggests that this programming model may operate as a form of precision dosing. Hypothetically, the cumulative and non-exhaustive nature of BST could promote an adaptive molecular milieu characterized by efficient anabolic signaling, improved redox regulation, and attenuated chronic activation of inflammatory mediators associated with aging muscle. Confirmation of this hypothesis requires clinical trials integrating functional outcomes with molecular, mitochondrial, and senescence-related biomarkers.

In oncology populations, the implementation of structured resistance training warrants additional clinical considerations. Older adults undergoing chemotherapy, radiotherapy, or endocrine therapy frequently present with cancer-related fatigue, anemia, peripheral neuropathy, cardiotoxicity, and reduced physiological reserve [121]. Therefore, the application of BST in this context should be individualized, clinically supervised, and adapted according to treatment phase and symptom burden. Contemporary exercise-oncology guidelines support the safety and feasibility of supervised resistance training in cancer survivors but emphasize careful dose progression and monitoring [122,123]. In the case of cancer patients, it is widely demonstrated that PA intervention reduces complications during chemotherapy or in the long term. Therefore, theoretically structured training should have clinical effects in this type of patient, as previously described by the authors [108].

## 7. Linking Exercise and Cellular Senescence to Cancer Outcomes

Epidemiological and interventional data linking physical activity to cancer outcomes can be broadly grouped into three domains: incidence and primary prevention, survival and recurrence, and patient-reported outcomes such as fatigue and quality of life [124,125]. However, it is important to clarify that the evidence discussed in this section derives predominantly from physical activity and exercise interventions in general, rather than from studies specifically designed using block strength training (BST). Therefore, BST should be interpreted here not as an ontologically validated intervention per se, but as a structured resistance training framework through which established exercise-induced adaptations may be organized and applied in older adults. Accordingly, potential applications of BST in oncology settings should be interpreted with caution, as current evidence does not include direct intervention studies in cancer populations. Therefore, such applications should be considered preliminary and hypothesis-generating rather than clinically established. Most mechanistic and interventional evidence cited derives from mixed-sex cohorts without consistent sex-stratified analyses. Therefore, while we highlight older women as a clinically relevant subgroup (frailty/menopause), the mechanistic framework is based largely on mixed-sex evidence due to their higher frailty burden and cancer-related vulnerability, the biological mechanisms discussed should be interpreted cautiously and not as women-specific. Future research should specifically investigate potential sex-related differences in senescence-related responses to structured resistance training.

Clinical epidemiological evidence associates PA with lower cancer incidence and mortality [126,127]. For example, active older women have about a 10–20% lower risk of breast cancer compared to their least active peers. Moreover, cancer survivors who exercise tend to have better survival and lower recurrence rates, as shown in breast and colorectal cancer survivors that added nutritional interventions [128]. There is sufficient evidence to conclude that specific doses of aerobic, combined aerobic plus resistance training, and/or only resistance training could improve common cancer-related health outcomes, including anxiety, depressive symptoms, fatigue, physical functioning, and health-related quality of life [123]. These full benefits are partly mediated by exercise-induced improvements in body composition, insulin sensitivity, and systemic inflammation that create a less favorable environment for cancer development. Within this framework, structured resistance training models such as BST may provide a way to implement these adaptations in a manner aligned with functional deficits associated with aging, while incorporating effort regulation and tolerability as central elements of the training process [22,108].

The rising incidence of cancer is intimately associated with increased lifespan and the growing proportion of older adults, with 64% of cancers diagnosed in people aged 60 and above. Mechanisms underlying aging include accumulation of somatic mutations, deficient DNA damage repair machinery, telomere shortening, enhanced genomic instability, epigenetic alterations [129].

The controlled exercise may reduce senescence-associated pro-tumorigenic signaling [130]. Clinical benefits such as cancer prevention or attenuation of progression are described in recent clinical trials. For example, lung cancer patients who engaged in PA had a 24% lower risk of cancer-specific death (HR: 0.76, 95% CI: 0.69–0.84), while colorectal cancer patients experienced a similar efficacy (HR: 0.71, 95% CI: 0.63–0.80). In skin cancer, PA was associated with a non-significant reduction in mortality (HR: 0.86, 95% CI: 0.71–1.05). These data are consistent with survival benefits of PA in cancer patients, particularly for breast, prostate, lung, and colorectal cancers [131]. Importantly, these findings should not be interpreted as evidence of BST-specific effects, but rather as part of the broader exercise literature from which BST may draw its conceptual rationale. In this context, for example, in colon cancer, exercise programs initiated soon after adjuvant chemotherapy for colon cancer resulted in significantly longer disease-free survival and findings consistent with longer overall survival [132]. The median age of the patients was 61 years (range, 19 to 84), 51% were women, 90% had stage III disease. Regarding outcome, disease-free survival was significantly longer in the exercise group than in the health-education group. Other randomized trials in older patients with cancer show that exercise interventions could be associated with significantly improved quality of Life and lower depression in older adults with cancer [133]. These findings suggest that health care and mechanistic protocols should focus on implementing PA interventions to improve outcomes in this prevalent population.

Concerning the link between senescence processes and effectiveness of PA in older patients, neither amount of PA can stop the biological aging process, there is evidence that regular exercise can minimize the physiological effects of an otherwise sedentary lifestyle and increase active life expectancy by limiting the development and progression of chronic disease and disabling conditions [134]. Since there is no single pathophysiological marker, consensus panels have been used to measure senescent cell burden and the effectiveness of the interventions, for example, PA.

Several proinflammatory biomarkers show reduced levels following high-intensity PA protocol. In this view, VEGFA, MMP7, and IL6 levels were most predictive of incident and persistent major mobility disability [135]. These results generate interest in a clinical trial to test the hypothesis that higher-level PA interventions reduce senescent cell burden and, correspondingly, circulate levels of senescence-related proteins. In this context, BST may be considered a potentially useful model to structure resistance training interventions targeting these processes; however, its specific effects on senescence-related biomarkers and cancer outcomes remain to be empirically established.

## 8. Limitations

Despite the theoretical, biological, and functional benefits of BST, its implementation in older adults with cancer faces critical limitations that must be considered. First, there are specific contraindications related to treatment toxicity, such as the presence of severe peripheral neuropathy or chemotherapy-induced cardiotoxicity, which can compromise safety during high-intensity exercise. Furthermore, there is marked heterogeneity in exercise response, where factors such as reduced anabolic sensitivity and age-related mitochondrial dysfunction can cause molecular and strength adaptations to vary significantly among individuals. Finally, poor adherence to exercise presents a major challenge, exacerbated by symptoms such as cancer-related fatigue and reduced physiological reserve, requiring highly individualized and closely monitored programs to ensure long-term sustainability.

## 9. Conclusions

For older adults, whose frailty, sarcopenia, and limited treatment tolerance critically shape prognosis, these exercise-induced benefits are particularly relevant. BST targets neuromuscular deficits, functional reserve, and putative senescence-related pathways. It may help shift individuals from a high-risk, low-reserve phenotype to a more resilient clinical profile. BST shows consistent benefits in physical performance, autonomy, and adherence. However, its biological effects remain speculative. Rather than a mechanistically validated intervention, BST should be seen as a structured and feasible model to deliver established exercise-induced benefits. Any influence on aging-related processes, including senescence or cancer-related outcomes, remains hypothetical and requires confirmation through studies that integrate molecular, functional, and clinical endpoints. Future studies are required to directly evaluate the mechanistic and clinical effects of BST on senescence-related pathways, in order to validate the translational framework proposed in this review. Therefore, advancing mechanistic and clinical research should be considered a primary priority to validate the translational potential of BST in this context.

## Figures and Tables

**Table 1:** Description of BST program. Adapted from Jofré-Saldía et al., 2025 [108].

Block Strength Training			
LOAD	FORCE PEAK	POWER PEAK	FORCE TOLERANCE
**Intensity**	10 RM	10 RM	12 RM
**Sets**	3	3	3
**Repetitions**	6 (10)	5 (10)	10 (12)
**%rep**	60%	50%	83%
**Level of Effort**	Low-to-Mod	Low-to-Mod	High
**Cadence**	1-1-1	MIV-1-1	1-1-2
**ROM**	full	full	full
**RPE**	≤7	≤7	8-9
**Rest between set**	1-min	2-min	2-min
**Rest between exe**	2-min	2-min	2-min
**Total duration**	~25-min	~40-min	~55-min

RM: Repetition Maximum, %rep: Percentage of repetitions, MIV: Maximal Intent Velocity, ROM: Range of Motion, RPE: Rating of Perceived Exertion.

## Data Availability

Data sharing is not applicable. No new data were created or analyzed in this study.

## Abbreviations

The following abbreviations are used in this manuscript:

AMPK	AMP-activated protein kinase
BST	Block Strength Training
CAT	Catalase
CCL2	Chemokine (C-C motif) ligand 2
CD8^+^ T	Cluster of differentiation 8 positive T lymphocyte
CRP	C-reactive protein
DART	Developmental Adaptations in Response to Training
DNA	Deoxyribonucleic acid
EGF	Epidermal growth factor
EMT	Epithelial–mesenchymal transition
FAPs	Fibro-adipogenic progenitors
GPx	Glutathione peroxidase
HGF	Hepatocyte growth factor
HMGB1	High-mobility group box 1
HR	Hazard ratio
IGF	Insulin-like growth factor
IL-1β	Interleukin 1 beta
IL-6	Interleukin 6
IL-8	Interleukin 8
IL-10	Interleukin 10
IL-1ra	Interleukin 1 receptor antagonist
MMP	Matrix metalloproteinases
NK	Natural killer
PA	Physical activity
p16^INK4a^	Cyclin-dependent kinase inhibitor 2a
p21^CIP1^	Cyclin-dependent kinase inhibitor 1A
Rb	Retinoblastoma protein
ROS	Reactive oxygen species
SASP	Senescence-associated secretory phenotype
SA-β-gal	Senescence-associated β-galactosidase
SANRA	Scale for the Assessment of Narrative Review Articles
SIRT1	Sirtuin 1
SOD	Superoxide dismutase
SPARC	Secreted protein acidic and rich in cysteine
TGF-β	Transforming growth factor beta
TNF-α	Tumor necrosis factor alpha
VEGF	Vascular endothelial growth factor
